# Supplementary material for: The mediation effect of attitude on the association between knowledge and practices toward air pollution among commercial drivers and traders in South-Western Ghana: A cross-sectional study
Source: PLoS One. 2026 Jan 13;21(1):e0329726. doi: 10.1371/journal.pone.0329726 (PMC12799002; doi:10.1371/journal.pone.0329726)
Supplement: S1 Table — (DOCX) [file pone.0329726.s001.docx]

S1 Table Protective measures used against air pollution among drivers and traders

| **Protective measures** | **Drivers, n (%)** | **Traders, n (%)** |
| --- | --- | --- |
| Wearing facemask | 317 (51.3) | 245 (62.5) |
| Put on hat | 268 (43.4) | 130 (33.3) |
| Put on helmet | 5(0.8) | 1(0.3) |
| Putting activated Carbon | 6(1.0) | 1(0.3) |
| Putting green plants | 10(1.6) | 9(2.3) |
| Use of air purifiers | 12 (1.9) | 5(1.3) |

n(%): frequency(percentage); column total
