# Supplementary material for: The mediation effect of attitude on the association between knowledge and practices toward air pollution among commercial drivers and traders in South-Western Ghana: A cross-sectional study
Source: PLoS One. 2026 Jan 13;21(1):e0329726. doi: 10.1371/journal.pone.0329726 (PMC12799002; doi:10.1371/journal.pone.0329726)
Supplement: Attachment — Submitted filename: Response to Reviewers.docx [file pone.0329726.s003.docx]

16^th^ October, 2025

**Re: Resubmission of manuscript: ID PONE-D-25-35486**

Dear Prof. Desai

**Thank you for the opportunity to revise our manuscript, “The mediation effect of attitude on the association between knowledge and practices toward air pollution among commercial drivers and traders in South-Western Ghana: a cross-sectional study”**

We appreciate the careful review and constructive suggestions. We have re-examined our work and made the required revisions as suggested. After making the required edits, it is our belief that the manuscript is improved. We have added the reviewer’s comments with our response in bold italics, including where and how our comments were modified. We have also marked changes in the manuscript using track changes. The revision of the manuscript was developed in consultation with all the co-authors, as the final edits is approved by all.

We have uploaded both the marked-up copy of our manuscript that highlights changes made to the original version as ‘Revised Manuscript with Track Changes’ and unmarked version of our revised paper without tracked changes as ‘Manuscript’

Thank you for considering our revisions

Sincerely yours,

Enoch Akyeampong

**Response to Editors Comments**

**Comment 1: Please ensure that your manuscript meets PLOS ONE's style requirements, including those for file naming**

Response 1: Thank you for this observation. We have revised our manuscript to meet PLOS One’s style requirement. The changes can be found in the upload named

**Comment 2:  Your ethics statement should only appear in the Methods section of your manuscript. If your ethics statement is written in any section besides the Methods, please move it to the Methods section and delete it from any other section. Please ensure that your ethics statement is included in your manuscript, as the ethics statement entered into the online submission form will not be published alongside your manuscript.**

Response 2: We have deleted the ethics statements from the declaration section our manuscript and maintained in the methods section only. Additionally, our ethics statement with corresponding approval credentials are included in our manuscript

**Comments 3: We note that Figure 1 in your submission contain [map/satellite] images which may be copyrighted. All PLOS content is published under the Creative Commons Attribution License (CC BY 4.0), which means that the manuscript, images, and Supporting Information files will be freely available online, and any third party is permitted to access, download, copy, distribute, and use these materials in any way, even commercially, with proper attribution. For these reasons, we cannot publish previously copyrighted maps or satellite images created using proprietary data, such as Google software (Google Maps, Street View, and Earth). For more information, see our copyright guidelines:** [**http://journals.plos.org/plosone/s/licenses-and-copyright**](http://journals.plos.org/plosone/s/licenses-and-copyright)**.**

**We require you to either (1) present written permission from the copyright holder to publish these figures specifically under the CC BY 4.0 license, or (2) remove the figures from your submission:**

**a. You may seek permission from the original copyright holder of Figure 1 to publish the content specifically under the CC BY 4.0 license.**

**We recommend that you contact the original copyright holder with the Content Permission Form (**[**http://journals.plos.org/plosone/s/file?id=7c09/content-permission-form.pdf**](http://journals.plos.org/plosone/s/file?id=7c09/content-permission-form.pdf)**) and the following text:**

**“I request permission for the open-access journal PLOS ONE to publish XXX under the Creative Commons Attribution License (CCAL) CC BY 4.0 (**[**http://creativecommons.org/licenses/by/4.0/**](http://creativecommons.org/licenses/by/4.0/)**). Please be aware that this license allows unrestricted use and distribution, even commercially, by third parties. Please reply and provide explicit written permission to publish XXX under a CC BY license and complete the attached form.”**

**Please upload the completed Content Permission Form or other proof of granted permissions as an "Other" file with your submission.**

**In the figure caption of the copyrighted figure, please include the following text: “Reprinted from [ref] under a CC BY license, with permission from [name of publisher], original copyright [original copyright year].”**

**b. If you are unable to obtain permission from the original copyright holder to publish these figures under the CC BY 4.0 license or if the copyright holder’s requirements are incompatible with the CC BY 4.0 license, please either i) remove the figure or ii) supply a replacement figure that complies with the CC BY 4.0 license. Please check copyright information on all replacement figures and update the figure caption with source information. If applicable, please specify in the figure caption text when a figure is similar but not identical to the original image and is therefore for illustrative purposes only.**

Response 3: We appreciate our observation and the options provided to ensure academic honesty. We were unable to obtain permission from the original copyright holder to publish the figure (map) under the CC BY license, hence, we opted to supply a replacement figure that complies with the CC BY 4.0 license. For our replaced map, we have included source information and declared that it for illustrative purposes only

**Comment 4: Thank you for providing this information. Could you please clarify what assets from CartoDB were used, if any? Are they free to access or were they purchased?**

Response 4: We accessed GIS maps. We used a free trial access; however, we have decided against citing CartoDB, citing only OpenStreetMap, which is free to use under an open license consistent with CC BY 4.0 license.

**Comment 5: Improve the figure quality and work upon all suggestions of reviewers.**

Response 5: The quality of the figures has been improved significantly and all suggestions from reviewers responded to in a systematic manner.

**Comment 6: Please confirm that all assets sourced from CartoDB have now been removed from your submission**.

Response 6: All maps and spatial figures in this manuscript were created with OpenStreetMap as the base map. No CartoDB sourced assets have been used in our submission.

**Authors response to Reviewers Comments**

Reviewer 1

**Comment 1: Improve language and figure quality**

Response 1: Thank you for taking time read our manuscript and the observation made with regards the quality of language. We have read the entire manuscript again and sought external review to ensure the language quality is improved. Additionally, we have improved the quality of our figures for clarity

**Comment 2: Compare your findings with existing standard reports as well as experimental data**

Response 2: We appreciate your observation and suggestion. We have improved the results presentation by not merely reporting the numbers but by evaluating them as have been reported elsewhere

**Comment 3: Inclusion-Exclusion criteria must be discussed in detail**

Response 3: Although the inclusion and exclusion criteria are clearly defined under study design and participants, we have delineated the inclusion and exclusion criteria and discussed in detail.

**Comment 4: Include AI based predictions from the dataset.**

Response 4: thank you for insightful suggestion to incorporate AI based predictions. This is an important and interesting idea for future research. However, our methodological scope is focused on establishing structural relationships and mediation pathways. Introducing AI based predictions could shift the focus of our study from explanation to prediction, an approach reserved for a subsequent study. We have a added a sentence future research to acknowledge this fascinating direction

Reviewer 2

This paper investigates a significant but less researched area by assessing the knowledge, attitudes, and practices (KAP) of commercial drivers and traders regarding air pollution in Ghana. The research is well-justified with a large sample size and regression and structural equation models (SEM) for mediation impact assessment. The ethical clearance and access to data have been well-discussed and thereby enhance transparency.

**Comment 1: The hypotheses at times conceptually blur (attitude → knowledge, etc.), and would be improved by more explicit justification or rewriting in accordance with traditional KAP/KAB frameworks.**

Response 1: Thank you for observation and suggestion. We acknowledge the error of the presentation of our findings, hence, we have corrected the error by rewriting in accordance with traditional KAP frameworks. In figure 5, therefore, it now reads knowledge	attitude.

**Comment 2: The modified questionnaire lacks psychometric validation (Cronbach's alpha, etc.), and so generates doubts concerning reliability.**

Response 2: We appreciate your suggestion about the psychometric validation. We have performed Cronbach alpha on the knowledge, attitude and practices. These variables showed strong reliability scores, for example, knowledge (a=0.79), attitude (a=0.70) and practices (a=0.78).

**Comment 3: Statistical reporting also has room for improvement: p-values should consistently be formatted.**

Response 3: Thank you for your observation and suggestion. We noticed that Table 2 did not report the p-values. We have corrected this by adding the p-values to ensuring consistent format.

**Comment 4: The analysis is repetitive of results instead of critically evaluating them, and would benefit from greater emphasis on policy implications, for instance, cheap protective gear or enforcement of emissions.**

Response 4: We take notice of this suggestion. We have improved the presentation of the results from repeating the findings to evaluating the findings with emphasis on policy implications

**Comment 5: The figures and tables similarly require more precise labels and presentation.**

Response 5: We have improved the clarity of the figures and improved the labels of both the Tables and Figures for clarity
